# MiR-4328 inhibits proliferation, metastasis and induces apoptosis in keloid fibroblasts by targeting BCL2 expression

**DOI:** 10.1515/biol-2020-0056

**Published:** 2020-09-01

**Authors:** Hongmei Tang, Qi Chen, Wenyuan Yu, Tianlan Zhao

**Affiliations:** Department of Plastic Surgery, The Second Affiliated Hospital of Suzhou University, 215000, Suzhou, China

**Keywords:** keloid, miR-4328, BCL2, proliferation, metastasis, apoptosis

## Abstract

Keloids are considered to be a type of benign tumor. MicroRNAs have been reported to be involved in the formation and growth of keloids. MicroRNA-4328 (miR-4328) was found to be abnormally expressed in keloids, while the role and the detailed molecular mechanism of miR-4328 in keloids remain unclear. The expression of miR-4328 and B-cell lymphoma 2 (BCL2) mRNA was detected by qRT-PCR. The proliferation, migration, invasion and apoptosis of keloid fibroblasts (KFs) was examined using Cell Counting Kit-8 assay, transwell assay or flow cytometry, respectively. Western blot was used to detect the level of proliferating cell nuclear antigen, cleaved-caspase 3, collagen I, collagen III and BCL2 protein. The interaction between miR-4328 and BCL2 was confirmed by luciferase reporter analyses. It was observed that miR-4328 was down-regulated in keloid tissues and fibroblasts, and miR-4328 restoration mediated the inhibition of proliferation, metastasis, collagen synthesis and the promotion of apoptosis in KFs. BCL2 was up-regulated in keloid tissues and fibroblasts, and BCL2 knockdown promoted the deterioration of KFs. In addition, BCL2 was confirmed to be a target of miR-4328, and the rescue experiment indicated that the inhibitory action of miR-4328 on keloid fibroblast progression was reversed by BCL2 overexpression. Thus, our results demonstrated that miR-4328 restrained the deterioration of KFs by targeting BCL2, which sheds new light on miR-4328 as a promising target for keloid development and therapeutic.

## 1 Introduction

Keloids, also known as connective tissue hyperplasia, are the result of excessive hyperplasia of fibroblasts and collagen caused by the continuous hyperactivity of collagen anabolism during wound healing [1]. Keloids often extend past the original injury boundaries with an invasive and sustained growth, which is quite similar to the immortal growth of tumors [2,3]. Surgical resection, radiation therapy and hormones are commonly used treatments, while all of them have poor therapeutic effect and are accompanied by recurrence [4]. As the etiopathogenesis of keloids remains unclear, it is urgent for us to systematically understand the underlying mechanisms of keloid formation in order to explore novel therapeutic targets.

MicroRNAs (miRNAs) are a class of endogenous, short non-coding and single-stranded RNAs approximately 22 nucleotides in length, which have been reported to have pivotal effects on various biological processes, such as cell growth, differentiation, proliferation, metastasis and apoptosis [5,6]. Recently, miRNAs that are involved in keloid formation and growth have been identified, and have differential expression in keloid tissues and keloid-derived fibroblasts [7]. Besides, miRNAs have also been characterized as novel regulators of cellular processes, including fibroblast proliferation and extracellular matrix (ECM) deposition, which are related to wound healing [8]. For example, Yao et al. determined that overexpression of miR-1224-5p led to the inhibition of proliferation, migration, invasion and promotion of apoptosis in keloid fibroblasts (KFs) [9]. Shi et al. indicated that miR-203 suppressed the proliferation, invasion and ECM production of KFs by repressing EGR1 and FGF2 expression [10]. Overexpression of miR-181a was also shown to enhance keloid fibroblast DNA synthesis, proliferation and inhibit apoptosis, thus inducing the progression of keloids [11]. Thus, miRNAs play important roles in fibrosis of keloids and may be a new target for gene therapy. Recently, microRNA-4328 (miR-4328), a member of the miRNA family, was first determined to cause aberrant expression in keloids [12]; however, the role and the exact molecular mechanisms of miR-4328 in keloids remain overlooked.

The B-cell lymphoma 2 (BCL2) gene, located on chromosome 18q21.3, encodes an integral outer mitochondrial membrane protein that blocks the apoptotic death of some cells, such as lymphocytes [13]. BCL2 protein belongs to the BCL2 family, which induces or inhibits apoptosis to determine the life cycle length of cells expressing the respective proteins [14]. In recent times, emerging evidence has revealed that BCL2 may be implicated in the growth of KFs [15,16].

In the present study, we explored the potential role of miR-4328 in keloids. We confirmed that BCL2 was a target of miR-4328 and that miR-4328 could inhibit the proliferation, metastasis, collagen synthesis and induce apoptosis in KFs by targeting BCL2, indicating a novel target for prevention and therapy of keloids.

## 2 Materials and methods

### 2.1 Fibroblast isolation and culture

Keloid tissues and normal skin tissues adjacent to the scar were obtained from 23 patients who underwent keloid excisional surgery at the Second Affiliated Hospital of Suzhou University. None of the patients had received systemic or topical treatment previously.

Keloid tissues (6 total) and adjacent normal tissues (6 total) were washed with phosphate-buffered saline (PBS) solution and cut into 1 mm^3^ pieces and then small tissue pieces were inoculated in the bottom of culture flasks at 37°C with 5% CO_2_. The fibroblasts grew out of tissues after 10–12 days of culturing. Then, primary KFs were gathered and cultured in Dulbecco’s Modified Eagle’s Medium (DMEM; Gibco, Rockville, MD) containing 10% fetal bovine serum (FBS, Gibco), 100 µg/mL of streptomycin (Sigma-Aldrich, St. Louis, MO, USA) and 100 U/mL of penicillin in a 5% CO_2_ incubator at 37°C. Subsequently, primary KFs were subcultured every 3 days using PBS/0.25% trypsin–ethylenediaminetetraacetic acid (EDTA). KFs from passage 3 to 8 were used for the experiment.


**Informed consent:** Informed consent has been obtained from all individuals included in this study.
**Ethical approval:** The research related to human use has been complied with all the relevant national regulations, institutional policies and in accordance with the tenets of the Helsinki Declaration, and has been approved by the Ethics Committee of the Second Affiliated Hospital of Suzhou University.

### 2.2 Cell transfection

miR-4328 mimic (miR-4328), mimic negative control (NC), miR-4328 inhibitor (anti-miR-4328), inhibitor negative control (anti-NC), pcDNA3.1-BCL2 overexpression vector (pc-BCL2), pcDNA3.1 empty vector (pcDNA), small interfering RNA (siRNA) against BCL2 (si-BCL2) and siRNA negative control (si-NC) were obtained from GenePharma (Shanghai, China). The synthetic oligonucleotides or vectors were transfected into KFs using Lipofectamine 2000 (Invitrogen, Waltham, MA, USA) following the manufacturer’s instructions. After transfection for 48 h, cells were collected for further experiments.

### 2.3 Quantitative real-time polymerase chain reaction (qRT-PCR)

Total RNA was extracted from the tissue samples using TRIzol reagent (Invitrogen, Carlsbad, CA, USA). For the miRNA, in the precipitation step, the isopropanol was added and then precipitated at −20°C for 6 h before centrifugation. According to the manufacturer’s protocol, moderate amounts of RNA samples were transcribed into cDNA using a miScript II Reverse Transcription Kit (Qiagen GmbH, Hilden, Germany). Then, equal amounts of cDNA synthesis were detected by qRT-PCR with SYBR Green I (Takara, Dalian, China) on the Applied Biosystems 7500 (ABI7500) system (Applied Biosystems, Foster City, CA, USA). All experiments were repeated three times independently. Fold changes were calculated via 2^−ΔΔCt^ method and U6 and β-actin were used as internal standards. The primer sequences were as follows: BCL2 forward, 5′-GACTTCGCCGAGATGTCCAG-3′, reverse, 5′-GTGCAGGTGCCGGTTCAGG-3′; miR-4328 forward, 5′-GCGAGCACAGAATTAATACGAC-3′, reverse, 5′-GCGAGCACAGAATTAATACGAC-3′; β-actin forward, 5′-GTCAGAAGGATTCCTATGTG-3′, reverse, 5′-AGGTCTCAAACATGATCTGG-3′; U6 forward, 5′-CTCGCTTCGGCAGCACA-3, reverse, 5′-AACGCTTCACGAATTTGCGT-3′.

### 2.4 Western blot

Total proteins from tissues and KFs were isolated by RIPA lysis buffer (Solarbio, Beijing, China) and the protein concentration was determined using BCA protein assay kit (Beyotime, Beijing, China). Equal amounts of proteins were subjected to 10% SDS-PAGE gel and transferred to PVDF membranes (Bio-Rad, Richmond, CA, USA). Then, after blocking with 5% non-fat dry milk, the membranes were incubated with primary antibodies proliferating cell nuclear antigen (PCNA), cleaved-caspase 3, collagen I and III and β-actin (CST, USA) at 4°C overnight. Subsequently, the membranes were incubated with HRP-conjugated secondary antibodies at room temperature for 2 h. Experiments were performed three times. Protein blots were visualized using an ECL western blotting detection kit (Beyotime).

### 2.5 Cell proliferation

Cell proliferation was detected using Cell Counting Kit-8 (CCK-8) assay (Dojindo Molecular Technologies, Japan). Briefly, cells were seeded at a concentration of 5,000 per well into 96-well plates after transfection and cultured at 37°C in triplicate. Then, 10 μL of CCK-8 solution was added to each well, which was mixed with 100 µL of DMEM containing FBS (10%) and then incubated for another 2 h at 37°C. Finally, the absorbance was measured at 450 nm using a microplate reader at 0, 24, 48 and 72 h.

### 2.6 Cell apoptosis

The apoptosis rate was measured by means of Annexin V-FITC/PI apoptosis detection kit (Beyotime), according to the manufacturer’s instructions. After transfection, KFs were resuspended in Annexin V binding buffer and then stained with Annexin V-FITC and propidium iodide (PI) for 15 min in the dark. Finally, the apoptotic cells were analyzed via flow cytometer (BD Biosciences, San Jose, CA, USA). The experiment was repeated three times.

### 2.7 Cell migration and invasion

Cell migration and invasion experiments were conducted using 24-well transwell filters (Cell Biolabs, Inc., San Diego, CA, USA) pre-coated with Matrigel or uncoated. In brief, transfected KFs at a density of 5 × 10^3^ cells/mL were seeded into an upper chamber containing 100 mL serum-free DMEM. Then, 500 μL of DMEM containing 10% FBS was added into the lower chamber. After incubation for 16 h (migration) or 24 h (invasion) at 37°C, the non-migrated or non-invaded cells in the upper chamber were removed gently, and the migrated and invaded cells were fixed and stained using 0.1% crystal violet containing formaldehyde for 30–60 min. Five randomly selected fields were pictured and the numbers of migrated and invaded cells were counted using an inverted microscope (Olympus, Shinjuku Monolith, Japan) (100×). The experiment was repeated three times.

### 2.8 Luciferase reporter assay

The BCL2 3′UTR wild-type (WT) or mutant (MUT) with miR-4328 binding sequences was amplified and cloned into the pGL3 luciferase reporter vector (Promega, Madison, WI, USA) to synthesize BCL2-WT or -MUT luciferase reporter vector (pGL3-BCL2-WT or -MUT). Then KFs were co-transfected with corresponding WT or MUT vectors and miR-4328 mimics or mimic control or anti-miR-4328 or anti-NC using Lipofectamine 2000 (Invitrogen). After 48 h of transfection, a dual-luciferase assay kit (Biotek Synergy, USA) was used to analyze luciferase activity. All experiments were repeated three times independently.

### 2.9 Statistical analysis

All analyses were performed with GraphPad Prism 7.0 (GraphPad Inc., San Diego, CA, USA). All experiments were repeated three times, and the data were expressed as the mean ± SD. The correlation analysis was analyzed by Spearman’s correlation. Statistical analyses were completed using Student’s *t* test or one-way ANOVA as appropriate. *P* values less than 0.05 were considered to be statistically significant.

## 3 Results

### 3.1 MiR-4328 inhibits proliferation, metastasis, collagen synthesis and induces apoptosis in KFs

**Figure 1:**
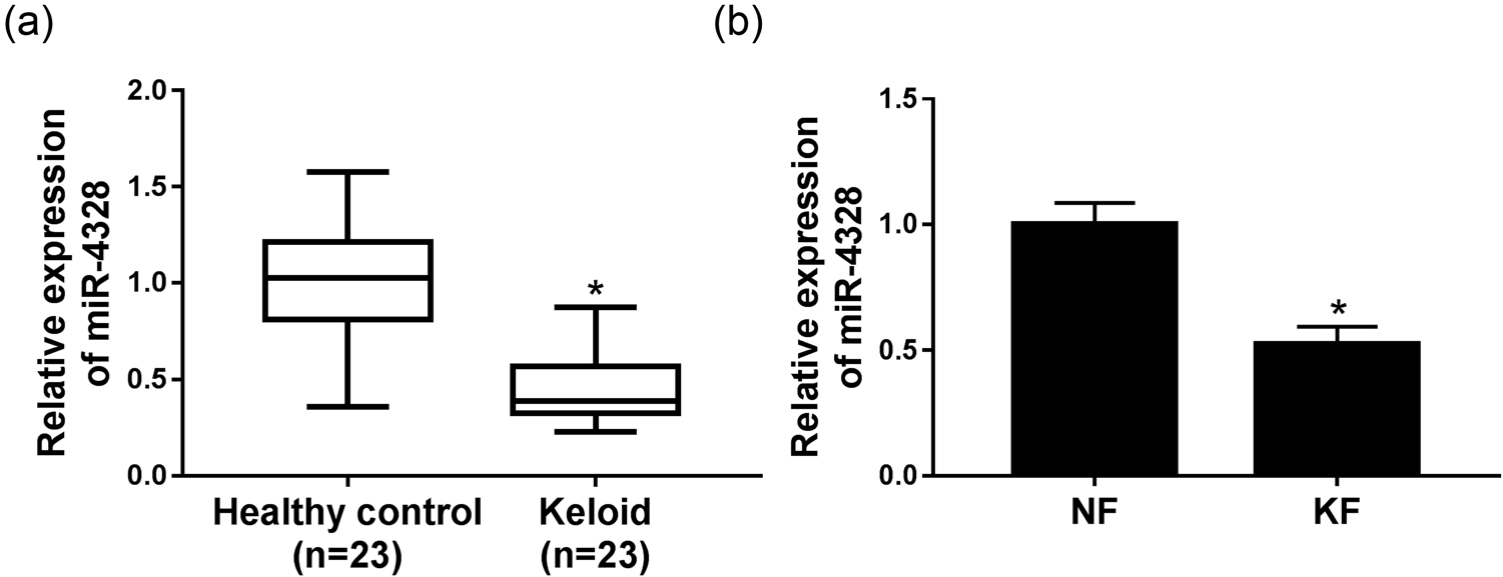
The expression of miR-4328 in keloid tissues and fibroblasts. (a) The relative expression of miR-4328 in keloid tissues and healthy skin tissues adjacent to the scar (control) obtained from 23 keloid patients was detected by qRT-PCR. (b) The relative expression of miR-4328 in fibroblasts derived from keloid tissues (6 total) and adjacent normal tissues (6 total) was detected by qRT-PCR. *P < 0.05.

**Figure 2:**
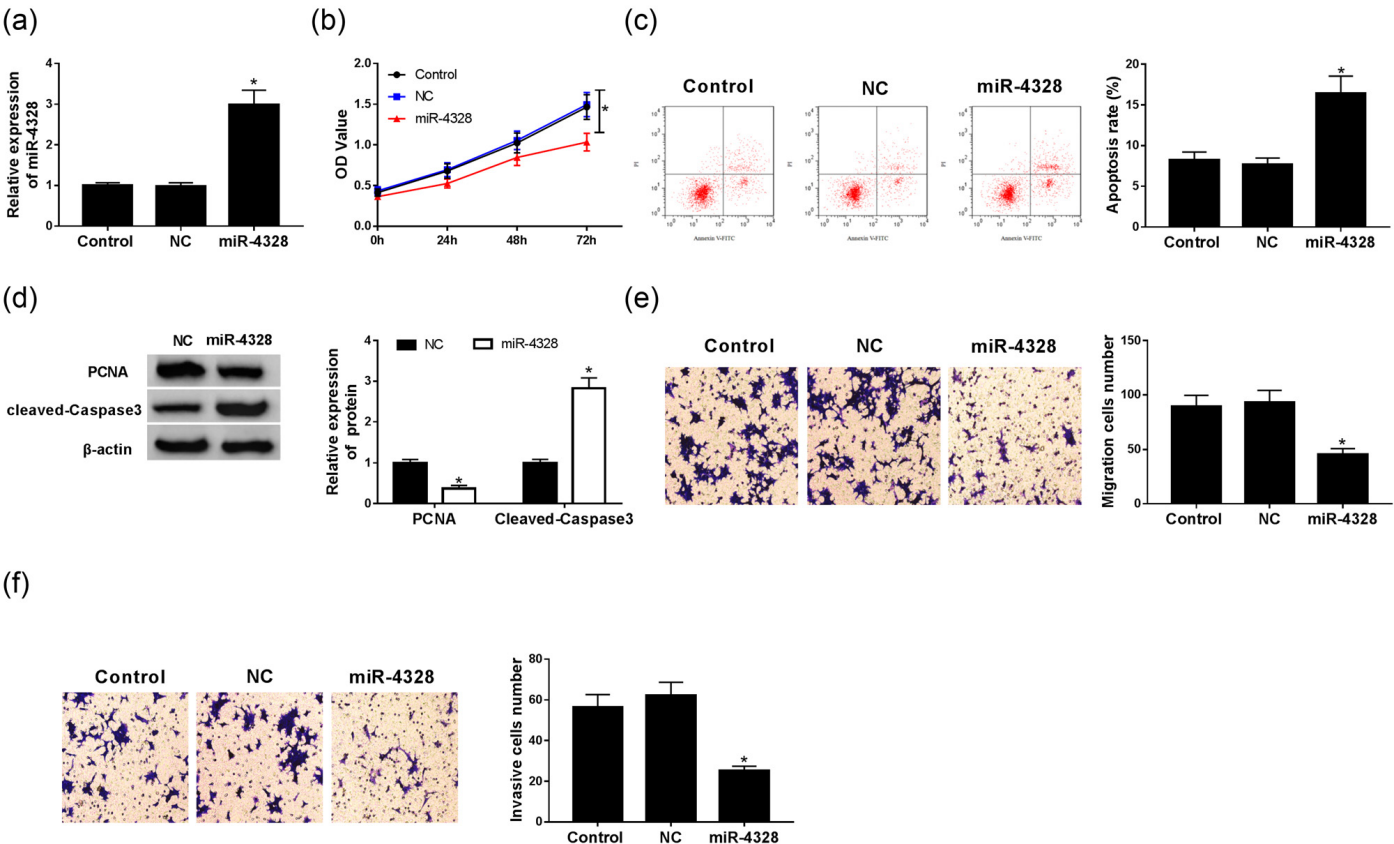
The effects of miR-4328 on keloid fibroblast progression. (a) The expression of miR-4328 was determined using qRT-PCR in KFs transfected with NC or miR-4328 mimic. (b) Cell proliferation was measured by CCK-8 assay. (c) Cell apoptosis rate was determined via flow cytometry. (d) Western blot was used to detect the protein of PCNA and cleaved-caspase 3. The migration (e) and invasion (f) abilities were evaluated using a transwell assay. *P < 0.05.

**Figure 3:**
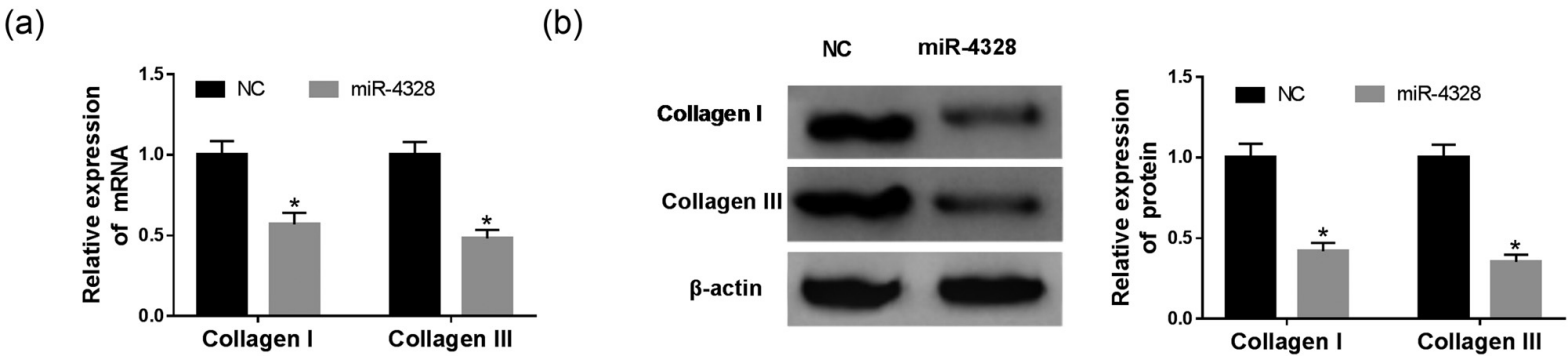
The expression of collagen I and III in KFs. (a) The expression of collagen I and III mRNA was investigated by qRT-PCR. (b) Western blot was used to explore the relative expression of the protein of collagen I and III. *P < 0.05.

The expression of miR-4328 was detected using qRT-PCR and the data showed a significant decrease of miR-4328 both in keloid tissues (Figure 1a) and fibroblasts (Figure 1b). Then in order to evaluate the biological effects of miR-4328 in human KFs, the keloid cells were transfected with a miR-4328 mimic, miR-NC, and an up-regulation of miR-4328 in KFs after transfection was found (Figure 2a). Subsequently, cell proliferation and metastasis abilities were detected and the results indicated that overexpression of miR-4328 depressed keloid fibroblast proliferation (Figure 2b), migration (Figure 2e) and invasion (Figure 2f). Meanwhile, cell apoptosis was measured by flow cytometry or western blot, and results showed that the apoptosis rate was greatly elevated in KFs (Figure 2c) and the western blot data suggested an inhibition of PCNA protein but enhancement of cleaved-caspase 3 protein (Figure 2d). Moreover, suppression of the expression of collagen I and collagen III mRNA (Figure 3a) and protein (Figure 3b) was validated. Therefore, all the data described herein verified that miR-4328 could inhibit proliferation, metastasis, collagen synthesis and induce apoptosis in KFs.

### 3.2 MiR-4328 directly targets BCL2 and suppresses BCL2 expression

**Figure 4:**
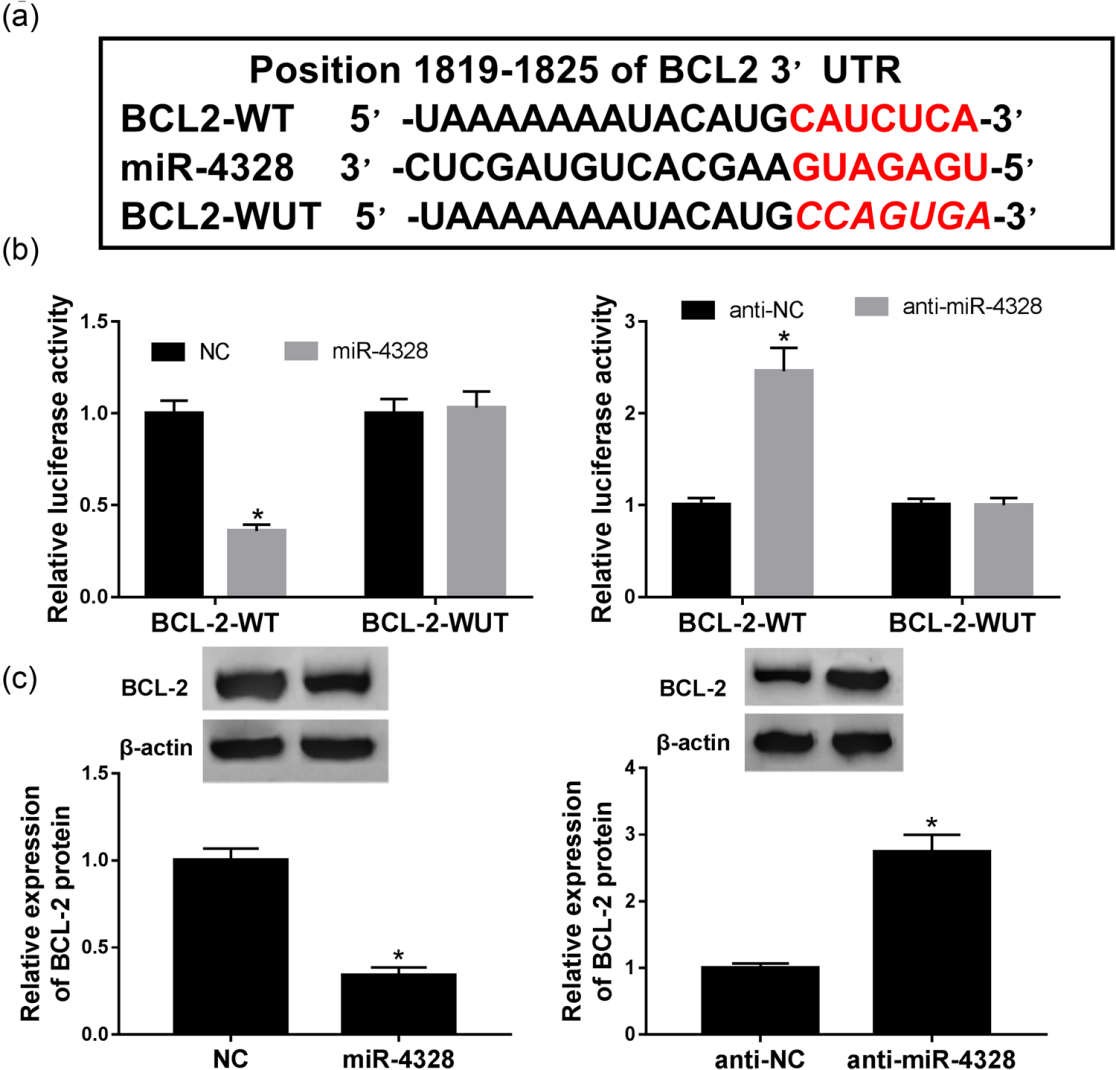
BCL2 was a target of miR-4328. (a) The putative binding sites of BCL2 3′-UTR and miR-4328. (b) Luciferase activity was measured in KFs co-transfected with BCL2-WT or BCL2-MUT and NC, miR-4328, anti-NC or anti-miR-4328. (c) The expression of BCL2 protein was examined in KFs transfected with NC, miR-4328, anti-NC or anti-miR-4328 by western blot. *P < 0.05.

To illustrate the underlying mechanism of miR-4328 involved in the progression of keloids, the potential targets were explored by bioinformatics analysis and the putative binding sites of miR-4328 and BCL2 are shown in Figure 4a. To confirm the hypothesis, luciferase reporters containing the miR-4328 binding site on the BCL2 3′UTR were constructed and we found that the miR-4328 mimic reduced the luciferase activity of the BCL2-WT reporter vector but not the BCL2-MUT reporter vector in KFs. While the miR-4328 inhibitor showed the opposite effect (Figure 4b). Additionally, the protein of BCL2 was measured, and miR-4328 mimic transfection inhibited BCL2 expression, while miR-4328 inhibitor enhanced BCL2 expression (Figure 4c). These results indicated that miR-4328 specifically suppressed BCL2 expression.

### 3.3 Knockdown of BCL2 inhibits proliferation, metastasis, collagen synthesis and induces apoptosis in KFs

**Figure 5:**
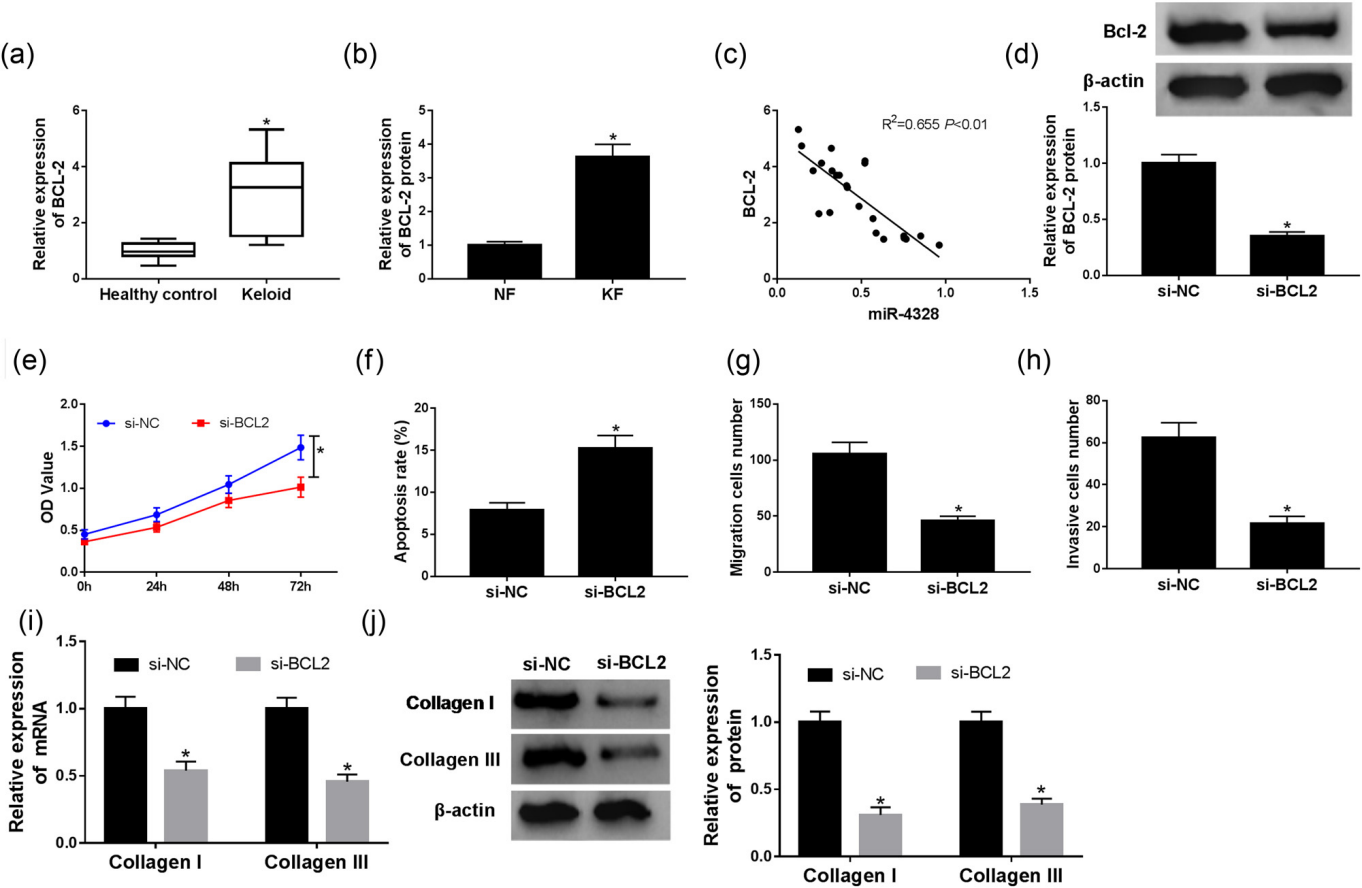
The effects of BCL2 on keloid fibroblast progression. (a) The relative expression of BCL2 in keloid tissues and normal healthy skin tissues adjacent to the scar (control) obtained from 23 keloid patients was detected by qRT-PCR. (b) The relative expression of BCL2 in fibroblasts derived from keloid tissues (6 total) and adjacent normal tissues (6 total) was detected by qRT-PCR. (c) The correlation between BCL2 and miR-4328 was analyzed by Spearman’s correlation analysis. (d) The transfection efficiency was detected in KFs transfected with si-BCL2 or si-NC. (e) Cell proliferation was measured via CCK-8 assay. (f) Cell apoptosis rate was determined by flow cytometry. The migration (g) and invasion (h) abilities were evaluated using a transwell assay. The expression of collagen I and III mRNA and protein was investigated by qRT-PCR (i) and western blot (j), respectively. *P < 0.05.

The expression of BCL2 was detected and we saw an obvious up-regulation of BCL2 protein both in keloid tissues (Figure 5a) and fibroblasts (Figure 5b). Furthermore, we discovered a significant negative correlation between BCL2 and miR-4328 expression (Figure 5c). To elucidate the effects of BCL2 on KFs, cells were transfected with si-BCL2 or si-NC, and the transfection efficiency was detected (Figure 5d). After transfection, proliferation, metastasis abilities and apoptosis rate of KFs were investigated and the data showed that knockdown of BCL2 inhibited proliferation, migration and invasion but promoted apoptosis in KFs (Figure 5e–h). In addition, we also found a notable suppression of collagen I and III mRNA and protein expression after si-BCL2 transfection (Figure 5i and j). Taken together, we determined that knockdown of BCL2 inhibited proliferation, metastasis, collagen synthesis and induced apoptosis in KFs.

### 3.4 MiR-4328 exerts inhibitory effects via targeting BCL2 expression in the deterioration of KFs

**Figure 6:**
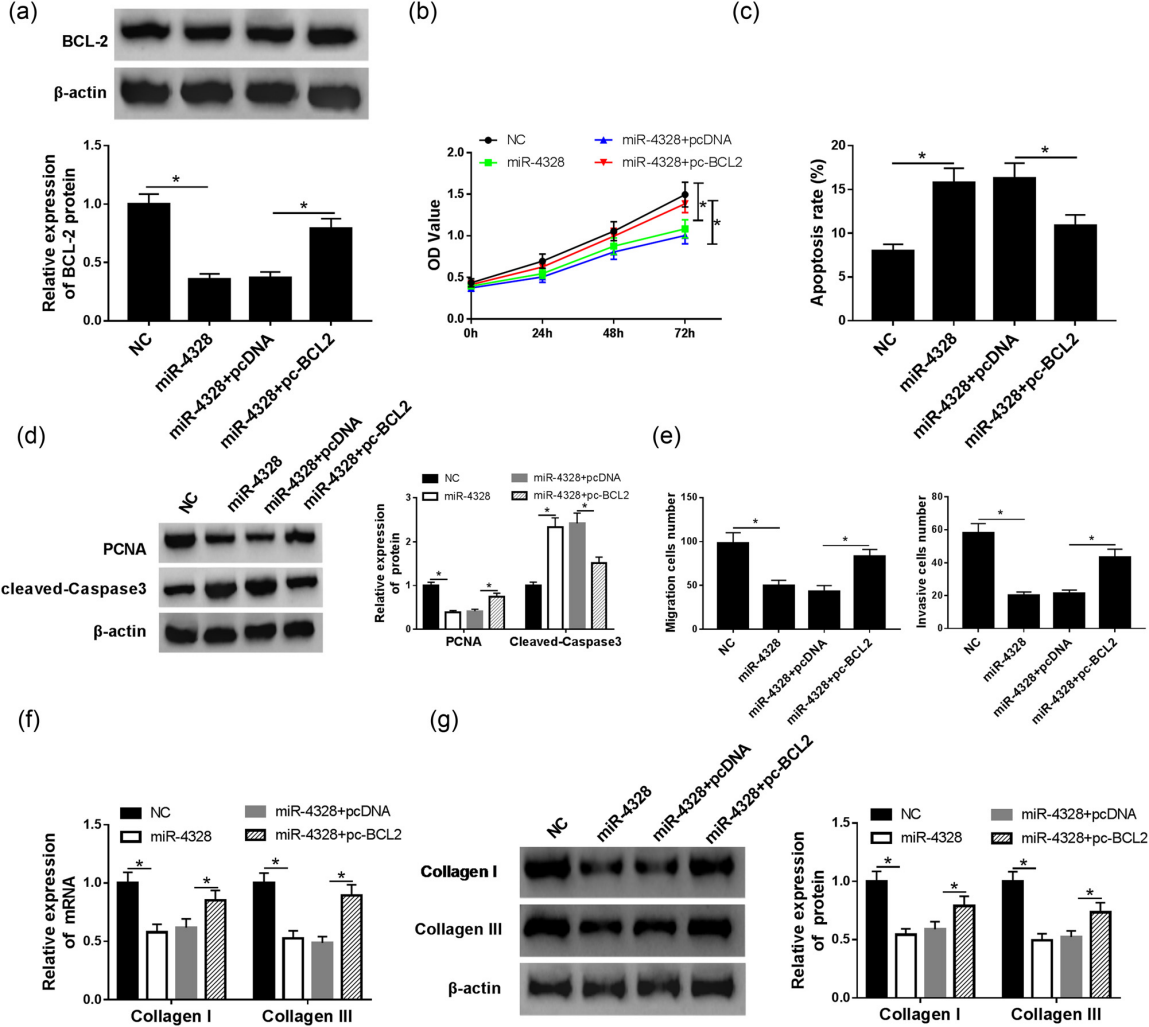
The interaction of miR-4328 and BCL2 on the progression of KFs. (a) The transfection efficiency was detected in KFs after transfection with miR-NC (NC), miR-4328, miR-4328 + pcDNA or miR-4328 + pc-BCL2. (b) Cell proliferation was measured via CCK-8 assay. Cell apoptosis rate was determined by flow cytometry (c) and western blot (d). (e) The migration and invasion abilities were examined via a transwell assay. The expression of collagen I and III mRNA and protein was evaluated by qRT-PCR (f) and western blot (g) in KFs. *P < 0.05.

Based on the above results, KFs were transfected with miR-NC (NC), miR-4328, miR-4328 + pcDNA or miR-4328 + pc-BCL2 to explore the interaction between miR-4328 and BCL2 on keloid fibroblast progression. After transfection, the protein expression of BCL2 was measured to verify the transfection efficiency (Figure 6a). Subsequently, keloid fibroblast proliferation, metastasis and apoptosis abilities were measured and the data indicated that miR-4328 mimic transfection inhibited proliferation (Figure 6b), migration and invasion (Figure 6e) but induced apoptosis (Figure 6c and d) in KFs, while pc-BCL2 transfection completely attenuated these effects. Additionally, the protein and mRNA expression of collagen I and III was evaluated via western blot and qRT-PCR, respectively. Results showed a depression of collagen I and III expression after miR-4328 transfection, while BCL2 overexpression counteracted the inhibitory effects of miR-4328 mimic induced on collagen I and III expression (Figure 6f and g). Therefore, we have demonstrated that miR-4328 repressed proliferation, metastasis, collagen synthesis and induced apoptosis in KFs via regulating BCL2 suppression.

## 4 Discussion

The excessive ECM components, such as collagen, fibronectin, elastin, proteoglycans, as well as matrix-directed protease and protease inhibitors, in keloids are caused by the accumulation of KFs. KFs play a vital role in pathological scars and their proliferation facilitates granulation tissue formation and leads to raise in collagen synthesis [17,18,19]. Unbalanced cellular dynamics caused by plethoric fibroblast proliferation and deficiency in fibroblast apoptosis results in keloids [12]. Excess deposition of ECM by fibroblasts is responsible for keloids, while the mechanism and etiology remain unclear. Recently, studies revealed that miRNAs could regulate fibroblast proliferation, metastasis, apoptosis and epithelial–mesenchymal transition (EMT) in keloid scarring. For example, down-regulation of miR-637 promoted proliferation and metastasis by targeting Smad3 in keloids [20]. MiR-152-5p inhibited proliferation, migration and promoted apoptosis by regulating the expression of Smad3 in human KFs [21]. MiR-21-5p linked EMT phenotype with stem-like cell signatures via AKT (protein kinase B) signaling in keloid keratinocytes [22]. In this study, we evaluated the role of miR-4328 in the progression of keloids. The expression of miR-4328 was detected using qRT-PCR and the data showed a significant decrease both in keloid tissues and fibroblasts. Additionally, previous studies reported that excess ECM components, such as collagen, were deposited by fibroblasts in keloids, indicating that fibroblasts participated in the etiology of keloids [23]. In the present study, the expression of collagen I and III was significantly decreased in keloid-derived fibroblasts after miR-4328 transfection, suggesting that miR-4328 repressed the formation of keloids. Subsequently, functionality experiments were performed and the data showed that a miR-4328 mimic transfection inhibited proliferation, metastasis and induced apoptosis in KFs. Then, in order to explore the underlying molecular mechanism, bioinformatics analysis was used to identify potential targets of miR-4328, and BCL2 was identified as a candidate target of miR-4328.

Apoptotic phenomenon regulation has been indicated to be important in arresting the wound healing process via cell apoptosis and has been primarily implicated in proliferation-cellular clearance [24]. Additionally, the apoptosis-suppressing protein BCL2 expression has been found scattered in both keloid and hypertrophic scar specimens, which together with the absence of p53 protein expression, could lead to cell proliferation and prolonged survival of cells in keloid-derived fibroblasts [25]. In addition, Bruce et al. found that BCL2 antagonist administration partially prevented fibrogenesis in intestinal fibroblasts and reduced the expression of TGF-β-induced factors involved in the differentiation of myofibroblasts; therefore, BCL2 might be a potential treatment option against Crohn’s disease-associated fibrosis [26]. Jian et al. revealed overexpression of miR-30a-5p induced keloid fibroblast apoptosis by targeting BCL2 [27]. All prior research indicates that BCL2 was associated with fibrogenesis. While the detailed mechanism of BCL2 in keloids was still ambiguous. In the present study, an obvious up-regulation of BCL2 protein both in keloid tissues and fibroblasts was detected, and knockdown of BCL2 inhibited proliferation, metastasis, collagen synthesis and induced apoptosis in KFs. In addition, the correlation between miR-4328 and BCL2 was confirmed and miR-4328 specifically suppressed BCL2 expression. Therefore, a rescue experiment was performed that further indicated that miR-4328 exerted the inhibitory effects through targeting BCL2 expression in KFs.

In conclusion, herein we have verified for the first time the effects of miR-4328 on the progression of keloids and have identified the miR-4328/BCL2 signaling pathway in KFs. These findings provide some support for the effective applications of miR-4328 as a novel target for prevention and therapy of keloids.
